# Unexplained Hyponatremia Revealing Vitamin D Deficiency: A Clinical Case Report

**DOI:** 10.1002/ccr3.71961

**Published:** 2026-01-31

**Authors:** Sara Yavuz Ileri

**Affiliations:** ^1^ Nephrology Department Ankara Gülhane Training and Research Hospital Ankara Türkiye

**Keywords:** hyponatremia, syndrome of inappropriate antidiuresis, vitamin D deficiency, vitamin D replacement

## Abstract

“SIAD” (syndrome of inappropriate antidiuresis) is frequently encountered in clinical practice, often necessitating the exclusion of various differential diagnoses and involving expensive treatments. In cases of SIAD concomitant with vitamin D deficiency, treatment with vitamin D supplementation may not only correct hyponatremia but also improve accompanying clinical findings, offering a cost‐effective alternative for clinicians. A 52‐year‐old Caucasian Turkish white male presented to the emergency department with symptoms of nausea, weakness, and altered consciousness. The patient was euvolemic. Laboratory results showed severe hyponatremia with low serum osmolality and high urine osmolality. Serum ADH levels were elevated. Extensive workup excluded other causes of SIAD. No significant pathology was detected, except for vitamin D deficiency. Following high‐dose vitamin D supplementation, the patient's sodium levels normalized without requiring further vasopressin receptor antagonist treatment. Long‐term follow‐up revealed that sodium levels remained within normal limits. Vitamin D deficiency is not traditionally considered among the common causes of SIAD. It may contribute to dysregulation of ADH. However, correcting this deficiency may resolve hyponatremia, making it a potentially overlooked, yet reversible, contributor. In patients with unexplained hyponatremia, vitamin D levels should be evaluated and corrected before initiating costlier treatment options.

## Key Clinical Message

Clinicians should consider checking vitamin D levels in patients with unexplained hyponatremia. Vitamin D replacement could be a simple and effective treatment, particularly in vitamin D–deficient individuals.

## 1 Introduction

The prevalence of hyponatremia in the emergency department (ED) is reported to range from 2.3% to 44% [1]. It is defined as a serum sodium level below 135 mmol/L. The balance of sodium levels in the body is primarily regulated by the interplay between water intake and excretion. Osmoreceptors in the hypothalamus sense plasma osmolality, leading to the secretion of antidiuretic hormone (ADH) from the posterior pituitary gland in response to increased plasma osmolality. ADH facilitates water reabsorption in the kidneys, resulting in the dilution of serum sodium levels.

Syndrome of Inappropriate Antidiuretic Hormone secretion (SIADH), more commonly known as SIAD, is characterized by the inappropriate release of ADH, which causes water retention despite low or normal plasma osmolality, ultimately resulting in euvolemic hyponatremia. Common causes of SIAD include malignancies (particularly small‐cell lung cancer), central nervous system disorders, pulmonary diseases, and certain medications.

Vitamin D deficiency is a widespread health issue that impacts various organ systems, including the skeletal, cardiovascular, and immune systems. Although the role of vitamin D in regulating ADH and fluid‐electrolyte balance is not yet fully understood, emerging evidence suggests a potential link. We present a case of SIAD with severe hyponatremia, where the only identifiable cause was vitamin D deficiency. Following vitamin D supplementation, the patient's sodium levels normalized, and long‐term follow‐up indicated that the patient remained normonatremic.

## 2 Case History

A 52‐year‐old Caucasian Turkish white male, with no known chronic disease and not on any regular medication, presented to the emergency department with nausea, general fatigue, and altered consciousness. He reported that he had been feeling unwell for several days. Physical examination showed that the patient was oriented to person but not to time or place, with no signs of dehydration or fluid overload. Vital signs were within normal limits.

Initial laboratory results revealed a serum sodium level of 121 mmol/L, serum osmolality of 270 mOsm/kg, urine osmolality of 566 mOsm/kg, and urine sodium of 120 mmol/L. These findings were consistent with SIADH. Thyroid and adrenal function tests were normal. Serum creatinine, calcium, phosphate, PTH, and liver function tests were within reference ranges. The patient had an ADH level of 3.6 pmol/L (reference range: 0.8–3.5 pmol/L), confirming inappropriately elevated ADH. However, ADH levels are notoriously unreliable. Despite elevated urine osmolality, urine sodium is 120 mmol/L.

## 3 Differential Diagnosis, Investigations, and Treatment

The patient was initially treated with 3% hypertonic saline and fluid restriction with 1000 mL on the first 8 h of hospitalization. Upon the absence of an increase in serum sodium levels and the persistence of clinical symptoms, Tolvaptan, a vasopressin receptor antagonist, was added due to persistent symptoms and inadequate sodium correction. Further workup included thoracic and abdominal imaging to rule out malignancies, especially small‐cell lung carcinoma. Chest CT revealed enlarged mediastinal lymph nodes with high FDG uptake on PET‐CT. Lymph node biopsy showed anthracofibrosis with no malignancy. After the addition of Tolvaptan, the patient's serum sodium level had increased from an initial value of 121–125 mEq/L, accompanied by a resolution of clinical symptoms at the 16th hour of hospitalization. On the second day, the serum sodium level reached 129 mEq/L, and the patient's hypertonic and Tolvaptan treatments were discontinued.

No other identifiable cause for SIAD was found. Meanwhile, vitamin D levels were checked and found to be severely low (25‐OH vitamin D: 10 nmol/L).

Therefore, high‐dose vitamin D supplementation (300,000 IU oral cholecalciferol monthly) was initiated. On the fourth day of hospitalization, after the patient's serum sodium level rose to 130 mEq/L, completion of the necessary tests and improvement of the clinical status, the patient was discharged under outpatient follow‐up.

## 4 Outcome and Follow‐Up

Within 2 weeks, the patient's serum sodium level increased to 134 mmol/L and the patient was maintained on weekly 50,000 IU cholecalciferol. During monthly follow‐up after discharge, the patient's serum sodium levels remained within the range of 140–142 mEq/L, and no clinical or laboratory evidence of recurrent hyponatremia was observed throughout the 3‐month follow‐up period; meanwhile, vitamin D levels were within normal limits (25‐OH vitamin D: 50 nmol/L; Table 1).

**TABLE 1:** Timeline of the clinical symptoms, treatments and laboratory findings throughout the patient's hospitalization and follow‐up period.

Date/time	Symptoms	Laboratory findings
Initial presentation	Patient presents with symptoms of hyponatremia	Serum sodium: 121 mmol/L
		Serum osmolality: 270 mOsm/kg
		Urine osmolality: 566 mOsm/kg
		Urine sodium: 120 mmol/L
		ADH level: 3.6 pmol/L (elevated)
		Normal thyroid and adrenal function tests
		Normal serum creatinine, calcium, phosphate, PTH, and liver function tests
First 8 h	Initiated treatment with 3% hypertonic saline and fluid restriction (1000 mL)	No increase in serum sodium levels noted
16 h post‐admission	Added Tolvaptan due to persistent symptoms and inadequate sodium correction	Serum sodium: 125 mEq/L
	Imaging studies performed to rule out malignancies	Chest CT: enlarged mediastinal lymph nodes with high FDG uptake
Day 2	Serum sodium level increased to 129 mEq/L; hypertonic saline and Tolvaptan treatments discontinued	Serum sodium: 129 mEq/L
	Vitamin D levels checked; found to be severely low	25‐OH vitamin D: 10 nmol/L
	Initiated high‐dose vitamin D supplementation (300,000 IU oral cholecalciferol monthly)	
Day 4	Patient's serum sodium level rose to 130 mEq/L; improvement in clinical status	Serum sodium: 130 mEq/L
	Patient discharged with outpatient follow‐up	
Two weeks post‐discharge	Serum sodium level increased to 134 mmol/L; maintained on weekly 50,000 IU cholecalciferol	Serum sodium: 134 mmol/L
Monthly follow‐up	Serum sodium levels remained within the range of 140–142 mEq/L; no clinical or laboratory evidence of recurrent hyponatremia	Vitamin D: 50 nmol/L (within normal limits)

## 5 Discussion

The presented case highlights a potential link between vitamin D deficiency and SIAD. In this patient, all common causes of SIAD were ruled out. Imaging and biopsy excluded malignancy, and there was no evidence of central nervous system (CNS), pulmonary, or drug‐induced causes. Notably, ADH was inappropriately elevated despite low serum osmolality. Remarkably, the only abnormal finding was a severe vitamin D deficiency, which, when corrected, led to normalization of sodium levels. But the spontaneous resolution of SIAD cannot also be excluded in this case.

The primary function of ADH is to maintain plasma tonicity. Osmoreceptors in the hypothalamus detect changes in effective plasma osmolality. A reduction in tonicity prevents ADH release, thereby averting water accumulation, while a rise in tonicity stimulates ADH release. ADH acts on V2 receptors located on the luminal surface of cortical and medullary collecting tubular cells. Under the influence of ADH, unique aquaporin‐2 water channels are formed by the fusion of pre‐formed cytoplasmic vesicles in the tubular cells, allowing water to be absorbed down the concentration gradient. Once water is absorbed, these channels are removed by endocytosis and returned to the cytoplasm. The osmotic threshold for ADH release in humans is approximately 280–290 mOsmol/kg, and osmoreceptors are extremely sensitive, responding to alterations in plasma tonicity as small as 1% [2].

When plasma osmolality is below this threshold, circulating ADH levels are minimal, leading to maximally diluted urine with an osmolality below 100 mOsmol/kg. Above the osmotic threshold, there is a relatively linear increase in ADH secretion. This regulatory system is so efficient that plasma osmolality typically does not vary by more than 1%–2% despite fluctuations in water intake [3].

In patients with SIAD, ADH levels are elevated even in the presence of decreased plasma osmolality and/or hyponatremia. This excess water absorption maintains blood volume at a high or normal level. While traditional causes of SIAD are well documented, emerging evidence suggests a potential role for vitamin D deficiency in its pathogenesis.

### 5.1 Vitamin D Deficiency and Its Impact

Vitamin D deficiency and insufficiency (defined as a level < 30 nmol/L and 30–50 nmol/L, respectively) are common even in countries with adequate sun exposure, with prevalence influenced by various factors including gender, nutrition, and mobility levels [4]. Recent large observational data have suggested that ~40% of Europeans are vitamin D deficient, and 13% are severely deficient [5].

Vitamin D deficiency is associated with a range of diseases, including immunological, cardiovascular, and musculoskeletal disorders, as well as hypertension, diabetes, and malignancy. Beyond its classical roles in calcium and bone metabolism, vitamin D has also been identified as a negative regulator of the renin‐angiotensin system (RAS).

Studies suggest that vitamin D suppresses renin gene expression, thereby modulating the renin‐angiotensin‐aldosterone system (RAAS) [6]. For instance, Li et al. demonstrated that 1,25‐dihydroxyvitamin D3 negatively regulates renin expression [7]. In vitamin D receptor (VDR) knockout mice, plasma renin and angiotensin II levels are significantly increased [8]. There is also evidence indicating that ADH levels are elevated in these mice, implying that vitamin D deficiency may lead to dysregulation of both RAAS and ADH.

However, research on the correlation between vitamin D deficiency and serum sodium imbalance remains limited. A connection between endogenous vitamin D synthesis and circulating sodium concentrations may be plausible but has not yet been definitively established. The exact pathophysiology underlying the observed association between these two biochemical impairments is still obscure.

Reports in the literature describe similar cases; for example, Gani et al. [9] documented an elderly patient with SIAD resistant to standard treatments who improved with vitamin D replacement. Another study by Pilz et al. [10] found an association between low vitamin D levels and hyponatremia in hospitalized patients. Both the presented case and previous reports highlight vitamin D deficiency as a significant factor in the pathogenesis of SIAD. In each instance, standard treatments for SIAD were ineffective until vitamin D supplementation was introduced, leading to improved sodium levels.

The mechanisms by which vitamin D deficiency contributes to SIAD may vary across cases. While some studies emphasize the role of vitamin D in modulating the RAAS, others suggest additional pathways that could be relevant. The severity of vitamin D deficiency and its impact on sodium levels may differ among patients, indicating a need for individualized assessment and treatment strategies.

Moreover, vitamin D significantly influences neuroendocrine regulation by modulating cortisol levels through hypothalamic–pituitary–adrenal (HPA) axis mechanisms [11]. Vitamin D receptors (VDR) are widely distributed in brain areas that control the HPA axis, including the hypothalamus and pituitary gland. It has been suggested that low vitamin D levels may contribute to abnormal cortisol secretion, while sufficient levels may help maintain balance in the HPA axis [12]. Glucocorticoids exert tonic suppression of ADH secretion, and hypocortisolism in secondary adrenocortical insufficiency can result in a clinical picture similar to SIAD [13]. This interaction implies that adequate levels of vitamin D may be necessary for optimal glucocorticoid receptor function, thereby influencing water balance and ADH secretion pathways.

In clinical practice, the standard management of SIAD includes fluid restriction, hypertonic saline, and vasopressin antagonists like Tolvaptan. These treatments can be costly and may have significant side effects. In contrast, vitamin D supplementation is safe, inexpensive, and widely accessible. Identifying and correcting vitamin D deficiency in patients with SIAD could prevent unnecessary interventions and reduce healthcare costs.

## 6 Conclusion

SIAD is a diagnosis of exclusion that often requires costly and invasive investigations. This case illustrates that vitamin D deficiency may be a reversible cause of SIAD. Clinicians should consider checking vitamin D levels in patients with unexplained hyponatremia. Vitamin D replacement could be a simple and effective treatment, particularly in vitamin D–deficient individuals. Further studies are needed to elucidate the exact mechanisms and establish guidelines for routine screening and treatment of vitamin D deficiency in hyponatremic patients.

## Author Contributions

**Sara Yavuz Ileri:** conceptualization, data curation, formal analysis, writing – original draft, writing – review and editing.

## Funding

The author has nothing to report.

## Ethics Statement

The author has nothing to report.

## Consent

Written informed consent was obtained from the patient for publication of this case report and any accompanying images. A copy of the written consent is available for review by the Editor‐in‐Chief of this journal.

## Conflicts of Interest

The author declares no conflicts of interest.

## Data Availability

Data supporting the findings of this study are available from the corresponding author on request.
